# Checkpoint Blockade Efficacy in Uveal Melanoma Is Linked to Tumor Immunity, CD28, and CCL8

**DOI:** 10.3390/ijms26209964

**Published:** 2025-10-13

**Authors:** Elias A. T. Koch, Renato Liguori, Alejandro Afonso Castro, Stefan Schliep, Anne Petzold, Anja Wessely, Waltraud Fröhlich, Fulvia Ferrazzi, Julio Vera, Markus Eckstein, Carola Berking, Markus V. Heppt

**Affiliations:** 1 Department of Dermatology, Deutsches Zentrum Immuntherapie (DZI), CCC Erlangen-EMN, Bavarian Cancer Research Center (BZKF), Uniklinikum Erlangen, Friedrich-Alexander-Universität Erlangen-Nürnberg (FAU), 91054 Erlangen, Germany; 2 Institute of Pathology, CCC Erlangen-EMN, Bavarian Cancer Research Center (BZKF), Uniklinikum Erlangen, Friedrich-Alexander-Universität Erlangen-Nürnberg (FAU), 91054 Erlangen, Germany; 3 Department of Nephropathology, Institute of Pathology, Friedrich-Alexander-Universität (FAU) Erlangen-Nürnberg, 91054 Erlangen, Germany; 4 Dermpath München, Laboratory for Dermatopathology, Oral Pathology, and Molecular Pathology, 80335 Munich, Germany

**Keywords:** uveal melanoma, immune checkpoint blockade, biomarkers, CD28, CCL8

## Abstract

For patients with metastatic uveal melanoma (UM), tebentafusp is currently the only systemic therapy approved by the EMA and FDA, but its use is limited to HLA-A*02:01-positive individuals. Immune checkpoint blockade (ICB) represents another option, though only a small subgroup of patients benefits, and no reliable predictive biomarkers are available to date. The aim of this study was therefore to identify parameters associated with favorable ICB response. Tumor samples and clinical data from 30 patients were analyzed. Group A (*n* = 16) showed clinical benefit, while Group B (*n* = 14) experienced disease progression. NanoString^®^ analyses revealed 258 upregulated genes in Group A, including IDO1, CD28, and CCL8. The enriched pathways were predominantly linked to immune activation, leukocyte adhesion, and responses to external stimuli. Immunohistochemistry confirmed significantly higher CD28 expression on infiltrating immune cells in Group A, while a machine learning approach identified CCL8 as a predictive marker with ~78% accuracy. Overall survival differed significantly between the groups. These findings indicate that patients responding to ICB display tumors with enhanced immune activation. CD28 and CCL8 emerged as promising candidates and should be validated in prospective studies to determine their clinical utility.

## 1. Introduction

Uveal melanoma (UM) is the most prevalent malignant eye tumor in adults, yet it is an orphan condition with an average incidence of 3–6 per million individuals in Europe [1]. Approximately 50% of patients, depending on the genetic composition of the primary tumor, develop metastases predominantly in the liver [2,3]. Once metastases are detected, the survival rate decreases rapidly from 52% after 1 year to 25% after 2 years and further to 13% after 3 years in the era before tebentafusp (tebe) and immune checkpoint blockade (ICB). For metastatic UM, tebe is the only treatment approved by the European Medicines Agency (EMA) and the Food and Drug Administration (FDA). The pivotal trial revealed a median overall survival (OS) of 21.6 months compared with 16.9 months in the control group, indicating that this drug is the first drug with a statistically significant survival benefit (HR 0.68; 95% CI, 0.54–0.87) [4,5]. Notably, this approach was evaluated against pembrolizumab, ipilimumab, or dacarbazine monotherapy, but not compared with double checkpoint blockade (DCB) [4]. Two published prospective single-arm trials investigated the use of DCB with ipilimumab and nivolumab in patients with metastatic UM. These trials reported differences in OS of 12.7 and 19.1 months for patients receiving DCB [6,7]. Furthermore, Petzold et al. undertook a broad meta-analysis of existing systemic treatments for metastatic UM, examining OS with a particular focus on tebe and DCB [8]. The median OS was 22.4 months for tebe, 15.7 months for DCB, 10.9 months for anti-PD-(L)-1 monotherapy, and 7.7 months for anti-CTLA-4 monotherapy. In a retrospective analysis, a good response to ICB, specifically a partial response (PR) according to the RECIST criteria, was shown to be a robust prognostic factor (*p* < 0.001) associated with prolonged OS, independent of DCB or ICB monotherapy [9]. A small subgroup of patients experiences survival advantages from ICB, yet the specific patient population that derives these benefits remains unidentified. Thus, the objective of this study was to identify parameters that are linked to a favorable response to ICB in patients with metastatic UM.

## 2. Results

### 2.1. Baseline Characteristics

**Figure 1:**
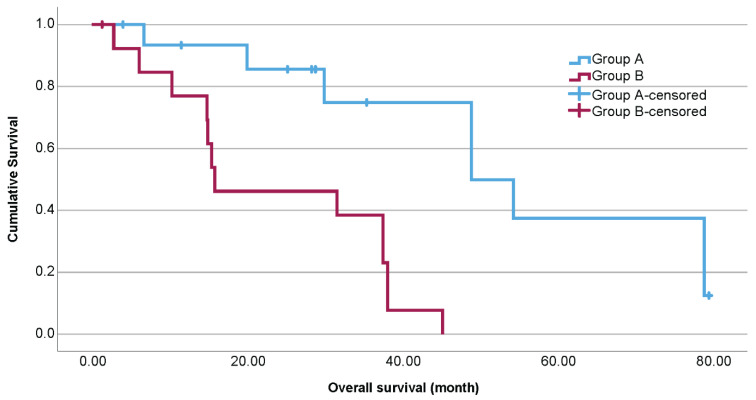
Kaplan–Meier estimates for overall survival (OS) according to therapeutic response to immune checkpoint blockade (group A = partial response (PR), stable disease (SD), and mixed response (MR); group B = progressive disease (PD)). The OS differed significantly (*p* < 0.001).

A total of 30 samples were included in the analysis. Among these patients, 26 received dual checkpoint blockade, and 4 received either anti-CTLA-4 or anti-PD-1 monotherapy. In this study, 14 patients had a PD (group B); 31% of the 16 patients in group A had a PR, 63% had an SD; and 6% had an MR to ICB. Baseline characteristics are summarized in Table 1. The estimated median OS of group A was 48.9 months (95% CI 27.3–70.4), and that of group B was 15.8 months (95% CI 0–35.3; Figure 1).

### 2.2. Differential Gene Expression

**Figure 2:**
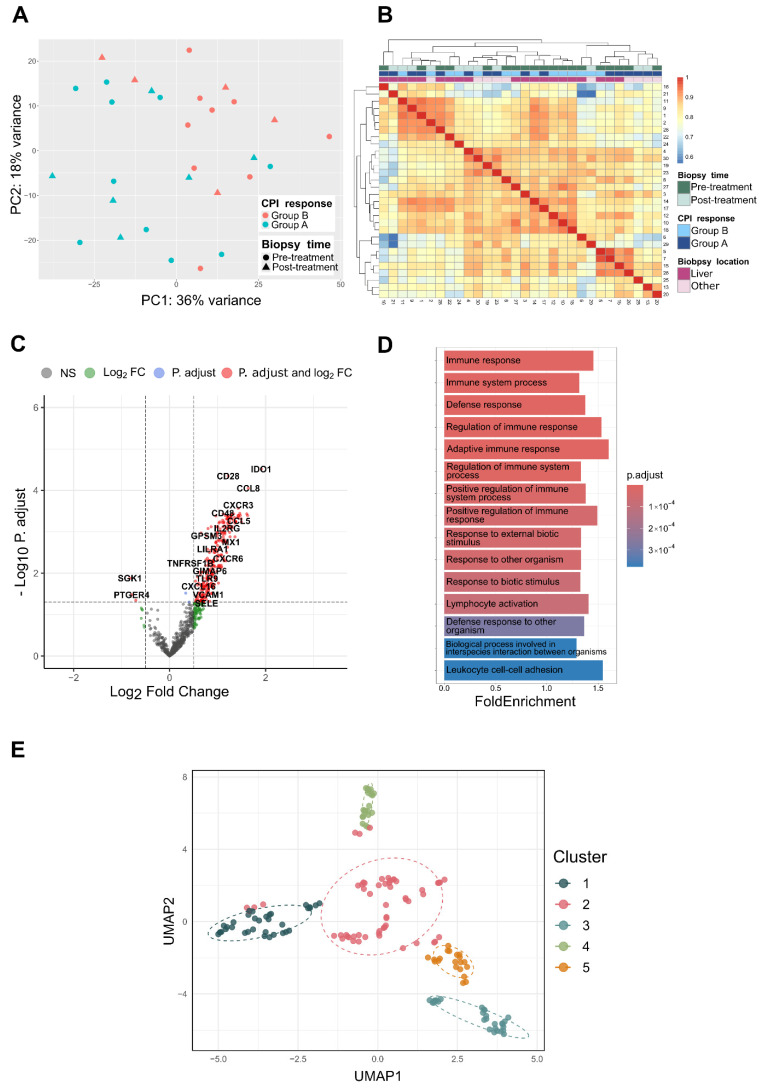
(**A**) Principal component analysis (PCA) plot of uveal melanoma samples based on the top 500 most highly expressed genes. The plot shows the first two principal components and the associated explained variance. The color corresponds to the ICB response group, and the shape corresponds to the biopsy time. (**B**) Heatmap of the pairwise Pearson correlations between samples. The color intensity reflects the strength of the correlation between samples; warmer colors (red) indicate greater similarity, and cooler colors (blue) indicate lower similarity. The annotation bars indicate the biopsy timepoint, ICB response group, and biopsy location. (**C**) Volcano plot of the differential gene expression analysis comparing group A (PR, SD, MR) to group B (PD). Genes are colored on the basis of the following cutoffs: log2-fold change (log2FC) > 0.5 and adjusted *p* value (P.adjust) < 0.05. Gray: nonsignificant genes (NS); green: genes with Log2FC > 0.5 only; blue points: genes with adjusted *p* value < 0.05 only; and red points: genes meeting both criteria (log2FC > 0.5 and adjusted *p* value < 0.05). (**D**) Bar plot showing the top 15 significantly overrepresented pathways. Pathways are ranked by adjusted *p* value (p.adjust) and colored accordingly, with warmer colors (red) indicating lower p.adjust values and cooler colors (blue) indicating higher p.adjust values. The X-axis displays the fold enrichment for each term. (**E**) UMAP (uniform manifold approximation and projection) visualization of similarities between the significantly enriched pathways. Each dot corresponds to a pathway, and the dot colors represent identified clusters.

The samples were evenly distributed throughout the Nanostring^®^ cartridges, and the principal component analysis (PCA) resulted in no significant clustering related to the sequencing runs (Figure S1). Instead, the dominant factor driving sample separation in the PCA was the response to ICB treatment (Figure 2A).

In the differential expression analysis, 258 differentially expressed genes (DEGs) were found between groups A and B (p.adj < 0.05). A total of 255 genes were upregulated in group A, and 3 genes were downregulated (Figure 2). The mean of the normalized counts and the *p* value distribution did not show any strange effects. The top three genes (*p* < 0.0001) were, in descending order, IDO1, CD28, and CCL8.

Analysis of T cell-associated genes revealed a consistent upregulation of several key markers. Subunits of the CD3 complex (CD3D/E/G; *p* = 0.008, 0.001, 0.001) as well as cytotoxic T cell markers (CD8A/B; both *p* = 0.001) and co-stimulatory molecules such as CD48 (*p* < 0.001) showed significantly elevated expression. Similarly, different CD45 isoforms (RA/RB/RO; *p* = 0.004, 0.001, 0.002) were increased, together with the activation markers CD69 (*p* = 0.002) and TNFRSF9/CD137 (*p* = 0.018). In contrast, the prototypical NK cell marker NCAM1/CD56 was unchanged. Nevertheless, activation-related genes shared by T and NK cells, including NCR1 (*p* = 0.029) and NKG7 (*p* = 0.003), were strongly induced. Beyond T and NK cell-specific markers, several molecules linked to antigen presentation and immune cell activation were also upregulated, including CD40L and CD40 (both *p* = 0.005), CD80 and CD28 (both *p* < 0.001), MRC1/CD206 (*p* = 0.03), as well as HLA-DRB1 (*p* = 0.013), HLA-DMA (*p* = 0.01), and HLA-DMB (*p* = 0.04). In contrast, beta-2-microglobulin showed no significant difference in expression (*p* = 0.15). Finally, the immune checkpoint receptors CTLA4 (*p* = 0.002) and PDCD1 (*p* < 0.001) displayed marked overexpression, highlighting enhanced activation and regulatory signaling within the T cell compartment. The full list of DEGs can be found in Supplementary Material S1.

To address the potential bias introduced by combining baseline (before treatment initiation) and longitudinal (during or after immune checkpoint blockade) tumor samples in the NanoString analysis, an additional control analysis was performed to disentangle the effects of biopsy timing. To assess the robustness of our findings, we re-analyzed the data by separating the cohorts according to biopsy timing. Specifically, we compared group A and group B within the baseline-only samples and within the longitudinal-only samples, thereby controlling for treatment timing as a potential confounder (Figure S2). The baseline samples showed results that were largely consistent with the main analysis (see list of DEGs provided in Supplementary Materials S2), whereas no DEGs were identified in the longitudinal samples, which further supports the validity and specificity of the main results.

### 2.3. Enrichment Analysis

Through overrepresentation analysis (ORA), 164 Gene Ontology (GO) biological process terms were significantly enriched in the differentially expressed genes (adjusted *p* value < 0.05). These pathways were predominantly involved in immune-related processes, including the regulation of the immune response, the activation of immune effector mechanisms, and the response to external stimuli (Figure 2D). Clustering-based identification of functional relationships between enriched terms revealed five clusters associated with distinct functional signatures (Figure 2E). Cluster 1 contained pathways related to immune activation, including terms such as ‘immune’, ‘positive’, ‘stimulus’, and ‘signaling’, capturing central processes in immune response activation. Cluster 2 was dominated by immune suppression and negative regulation, with terms such as ‘mediated’, ‘negative’, and ‘leukocyte’, suggesting involvement in regulatory mechanisms that may distinguish responders from nonresponders. Cluster 3 featured pathways associated with immune cell behavior, particularly cell activation, adhesion, and proliferation, indicating the modulation of immune cell dynamics. Cluster 4 grouped pathways involved in cell migration and chemotaxis, highlighting roles in lymphocyte trafficking and chemokine signaling. Cluster 5 included pathways related to cell differentiation and apoptosis, such as activation, differentiation, and apoptotic processes, suggesting that transcriptional programs are associated with immune cell fate decisions.

### 2.4. Machine Learning Pipelines

**Figure 3:**
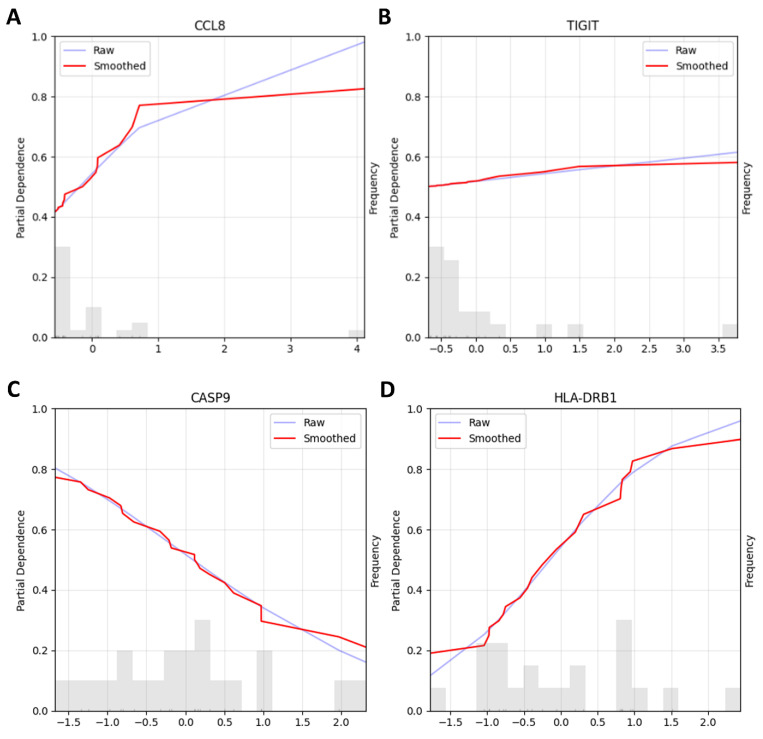
This figure shows two partial dependence plots (PDPs) illustrating the relationship between gene expression levels and the model’s predicted output. We used either a preselected subset of genes (top 5 genes, **A**,**B**) or all genes (**C**,**D**) and fed them into recursive feature selection for finer gene subselections. The best-performing pipeline with preselected genes selected CCL8 (**A**) and TIGIT (**B**) as biomarkers for use in the predictions and achieved ~78% accuracy on the testing dataset. Each plot shows both the raw (blue line) and smoothed (red line) partial dependence values, providing insight into how variations in gene expression affect the model’s predictions. Panel A, the plot for CCL8, shows that increasing expression levels are associated with an increasing predicted probability of stable disease/response to therapy, particularly at lower expression levels, where the curve shows a steeper slope. In Panel B, the TIGIT plot indicates that increasing TIGIT expression shows a modest linear effect on the predicted probability of stable disease/response to therapy. Below both plots, histograms display the distribution of gene expression levels in the dataset. While TIGIT was differentially expressed and was selected by the machine learning pipeline to be one of the genes used for prediction, it remains unclear whether its use as a biomarker for good clinical outcomes extends beyond our current dataset given the small sample size (*n* = 30), the moderate effects on the prediction outcome, and the fact that we failed to see the significance of TIGIT with an IHC stain, which we later performed. In the pipelines that used all genes, our best-performing one selected two genes for its prediction: CASP9 (**C**) and HLA-DRB1 (**D**). Panel C shows that increasing CASP9 expression is associated with a decreased predicted probability of stable disease/response to therapy. Panel D shows that increasing HLA-DRB1 expression is associated with an increased predicted probability of stable disease/response to therapy. The model achieved ~89% accuracy on the testing dataset. While CASP9 was not among the list of differentially expressed genes, HLA-DRB1 was, providing independent attestation to its possible value as a marker of therapy responders.

We conducted an exploratory machine learning analysis to examine gene expression patterns from a complementary descriptive perspective to support our primary differential expression analysis, since our capacity for developing validated predictive models was limited by a small sample size (*n* = 30). Two exploratory approaches were pursued:

First, to examine genes of primary interest from our differential expression analysis, we used the top 5 DEGs (IDO1, CD28, CCL8, CXCR3, and TIGIT, based on lowest adjusted *p*-values) as candidate features. The dataset was split into training and testing sets following a 70/30 stratified split, and further feature selection was performed on the training set with Recursive Feature Elimination (RFE) in order to boil down the number of features from 5 to 2 [10,11]. We desired to have two features since the size of the training set was 21, and a usual rule of thumb indicates a ratio of 1-to-10 features per sample. To explore both linear and non-linear feature selection patterns, two models were used for RFE: Logistic Regression and Gradient Boosting Classifier, with results from both approaches evaluated [12]. After the feature selection, seven models were compared through stratified 3-fold cross-validation on the training dataset according to their accuracy (see Supplementary Materials S3 for the full list of models evaluated). The best-performing model across all folds in terms of average accuracy was then trained on the whole training dataset and tested on the testing set. We report here the better-performing configuration: a Logistic Classifier that used the genes selected by the RFE with a Gradient Boosting Classifier. The RFE selected CCL8 and TIGIT among the 5 pre-selected genes, and the Logistic Classifier achieved ~78% accuracy on the held-out test set. Note that this approach involved look-ahead bias (data leakage), as the differential expression analysis that had served as the basis of gene selection used the full dataset (Figure 3A,B).

Second, to avoid the look-ahead bias, we performed feature selection exclusively on the training data without prior gene filtering, following standard machine learning methodology. Following the same steps as described above, we compared the same two RFE approaches, Logistic Regression and Gradient Boosting Classifier. The best-performing model was a Logistic Regression Classifier, which achieved an ~89% accuracy on the testing dataset. The genes used for this model were CASP9 and HLA-DRB1, which were selected through RFE with a Logistic Classifier (Figure 3 C,D).

These exploratory analyses were designed to complement our primary statistical findings by examining gene expression patterns through a machine learning lens. While these results suggest potential biomarker candidates, the small sample size and the descriptive nature of this analysis preclude definitive conclusions about predictive performance. Detailed methodology and additional performance metrics are provided in Supplementary Materials S3.

### 2.5. Immunohistochemistry

The mean immunoreactive score (IRS) of CD28 on infiltrating immune cells was 2.52 (±2.09 SD) in Group A and 1.2 (±1.79 SD) in Group B (*p* = 0.039). The expression of IDO1 and TIGIT did not differ significantly. For further information, see Table 2.

## 3. Discussion

In metastatic UM, the identification and characterization of “hot” tumors through reliable biomarkers is critical for selecting patients who may benefit from ICB, particularly given the lack of alternative treatment options and the low clinical benefit coupled with the risk of severe immune-related adverse events [13]. The presence and extent of biomarkers indicative of response to ICB vary between different cancer types and across treatment settings. The most widely validated and used biomarker to guide the selection of patients to receive anti-PD-1 or anti-PD-L1 antibodies is currently PD-L1, quantified using immunohistochemistry assays [14,15]. Several studies have indicated that elevated PD-L1 expression levels are associated with increased response rates and improved survival outcomes in a number of tumor types [16]. Nevertheless, the predictive capacity of PD-L1 is limited, with therapeutic responses occurring in certain PD-L1-negative tumors and a lack of benefit seen in some PD-L1-positive cases [14,17]. In UM tumors, the presence of PD-1+ infiltrates and PD-L1+ tumor cells was found to be minimal to non-existent [18]. A study indicated that the expression of PD-1 and PD-L1 in tumor-infiltrating lymphocytes (TILs) was associated with unfavorable clinical outcomes [19]. These findings suggest that these markers may aid in identifying a subgroup of UM patients who could potentially benefit from immunotherapy [19]. Further, tumor mutational burden (TMB) has been proposed as a predictive biomarker in several cancer types, including melanoma and lung cancer [20]. Notably, UM exhibits an exceptionally low TMB, with the lowest median among the 20 cancer types in a cohort of 6035 patients [21]. This finding suggests that TMB is unlikely to capture the immune-inhibitory biology of UM. Additional research has expanded the predictive landscape of ICB responsiveness beyond bulk transcriptomic signatures by incorporating tumor microenvironment heterogeneity and cell-level immune states. For instance, a TME-based classification in adrenocortical carcinoma highlighted the prognostic significance of immune infiltration patterns and stromal composition in determining therapeutic outcomes [22]. Similarly, an immunosubtyping framework integrating single-cell transcriptomics in hepatocellular carcinoma demonstrated that distinct immune cell subsets, such as NK cells, can function as pivotal determinants of ICB response [23].

Nevertheless, our analysis revealed that patients with a good response to ICB (group A) benefit with improved OS, as shown in our previous multicenter analyses [9]. Group A contains 258 DEGs, all of which are associated with the immune response, positive regulation of the immune process, leukocyte adhesion and activation, and the response to external stimuli. This implies that patients with a preexisting immune response against their tumor benefit the most from ICB, and these tumors may belong to the category of “hot” tumors. In solid tumors, responders exhibit an immune-inflamed phenotype referred to as “hot” tumors, whereas nonresponders are more prone to a cold, immune-excluded phenotype [24]. Generally, the immune phenotype is characterized by the infiltration of T cells in the tumor microenvironment, which may refer to TILs. We also observed increased expression of T cells and T cell activation markers in group A, which may indicate increased infiltration of T cells. However, in colorectal and ovarian cancer, for which T cell infiltrates are a positive prognostic marker, only approximately 10% of intratumoral CD8+ T cells have the capacity to recognize autologous tumor cells. Furthermore, some tumors exhibit no tumor-reactive T cells despite the presence of TILs [25]. The role of these bystander T cells in tumor immunology remains unknown. Sun et al. analyzed NGS data from UM samples and reported that low-risk patients had a better prognosis and responded better to ICB treatment [26]. These patients exhibited increased immune cell infiltration within the tumor, reduced numbers of immunosuppressive cells, enhanced activation of immune response pathways, and a higher immunophenoscore, consistent with our findings [26]. Qin et al. analyzed the expression of CD3, CD8, FoxP3, CD68, PD-1, and PD-L1 by IHC in 27 primary and 31 metastatic UM tumors and performed NanoString analyses of pre- and posttreatment samples from only 6 patients [18]. They reported no organ-specific differences in immune infiltrates, and the levels of PD-1+ and PD-L1+ were low to absent in all samples [18]. In the NanoString analyses, a suppressor of cytokine signaling, 1, was identified, potentially contributing to the response to immunotherapy. Furthermore, the expression of proinflammatory cytokines and molecules was significantly greater in tumors before ICB treatment, which is also in line with our results. However, they used a customizable gene expression panel (which has a low overlap with our panel) as well as a low number of samples. We used a panel designed for standardized immune profiling in cancer research, containing preselected genes focused on the immune response and tumor-immune interactions [27]. In our dataset, the top three genes that were notably significantly upregulated in ICB responders were CCL8, IDO1, and CD28. Through IHC validation, we detected significant overexpression of CD28 on infiltrating immune cells in group A, suggesting its potential as a marker for assessing responders. CD28 is expressed on the cell surface of naive CD4+ and CD8+ T cells and provides an essential costimulatory signal for T cell proliferation, cytokine production, and the promotion of T cell survival upon ligation by B7-1 and B7-2 on antigen-presenting cells [28]. Upon T cell activation, they express CTLA-4, which downregulates the immune response. As CTLA-4 levels increase, CD28 is reduced through endocytosis [28]. B7-2 is always present at low levels, whereas B7-1 is produced only when APCs detect infection, stress, or cell damage [29]. When activated, APCs increase the production of B7-1 and B7-2, which can either stimulate or regulate T cell activity [29]. This balance between the CTLA-4, CD28, and B7 proteins ensures that T cells can fight infections and cancer while preventing excessive or harmful immune responses, such as autoimmune diseases. On the other hand, CD28 in cancer cells (not T cells) promotes immune escape rather than immune activation. Recently, Yang et al. reported that knocking out CD28 in cancer cells (triple-negative breast cancer) increased the infiltration of dendritic cells (cDC1s) and activated CD8+ T cells, which are crucial for antitumor immunity [30]. Furthermore, CD28 in cancer cells promotes PD-L1 expression, which suppresses T cell activity [30]. However, in our IHC analysis, we detected CD28 expression only on immune infiltrates and not on tumor cells, which is consistent with previous observations of its essential role in T cell growth and survival. Nevertheless, for further investigation of CD28 as a biomarker for therapy response prediction in UM, the exact discrimination of CD28-expressing cells might be crucial. Despite its value as a biomarker, CD28 is an interesting target for immunotherapy. Notably, an anti-CD28 antibody (TGN1412) triggered a severe cytokine storm, resulting in life-threatening complications, 90 min after administration in a phase I trial [31]. Beyond classical antibody targeting, a potential strategy for modulating immune responses is to target the internal regulation of CD28 by interfering with its endocytosis. This approach could prevent or slow the internalization of CD28 from T cell surfaces. This could maintain the costimulatory function of CD28 even in the presence of high CTLA-4 levels [28]. Further, it was shown that SNX9 promotes CD28 clustering and enhances IL-2 production [32]. The enhancement of the CD28–SNX9 interaction may be beneficial in the context of immunotherapy.

IDO was one of the three top upregulated genes in group A. IDO levels are very distinct across different cancer entities and have different associations with overall prognosis. For example, a higher IDO transcript level correlated with reduced OS in glioblastoma patients, which is not the case in CM. In CM, the opposite is true: increased mRNA levels are correlated with improved OS [33,34]. However, in our study, it was not directly associated with improved survival and was more prone to a good response to ICB. IDO has also been used as a therapeutic target with IDO enzyme inhibitors and vaccines [35,36]. In particular, combination with ICB has already shown clinical efficacy in different tumor types [37,38]. Furthermore, preclinical studies have shown that IDO expression in cancer may contribute to the suppression of tumor immunity [33]. In that case, IDO might have been upregulated as a consequence of the immune response. However, IDO IHC expression was low or absent in our samples.

CCL8 has chemotactic effects on monocytes, lymphocytes, basophils, and eosinophils. Through the recruitment of leukocytes to inflammatory sites, this cytokine may contribute to tumor-associated leukocyte infiltration [39]. Chen et al. showed that blocking the AXL/MERTK signaling pathway reactivates antitumor immunity and sensitizes tumors to anti-PD-1 treatment [40]. Furthermore, CCL8 appears to reduce Treg infiltration into tumors, thereby lowering immunosuppression [40]. In large B cell lymphoma (LBCL), CCL8 was identified as a hub gene, which is involved in several immune activities and correlates with tumor-associated macrophages (TAMs) [41]. TAMs are known to suppress T cells, promote tumor growth, and upregulate checkpoint molecules such as PD-L1 [42]. These findings suggest that CCL8 plays a key role in increasing the response of the tumor microenvironment to immunotherapy. However, most of the currently available evidence in melanoma and other solid tumors is associative, and functional data demonstrating a causal role for CCL8 in modulating antitumor immunity are limited and sometimes contradictory. Some studies also report that CCL8 promotes the recruitment of immunosuppressive myeloid populations and supports tumor progression [43]. The divergent observations highlight the need for dedicated mechanistic studies before firm conclusions about its prognostic relevance can be drawn.

Given the exploratory nature of our findings, CD28 and CCL8 should currently be regarded as investigational markers that require analytical and clinical validation before they can be considered as biomarkers. While CD28 could be feasibly assessed by low-cost immunohistochemistry on routinely processed FFPE samples, the implementation of CCL8 remains challenging due to the lack of standardized and available protein-based assays.

One limitation of this study is that we cannot distinguish between host-derived and tumor-derived signals (only in CD28 expression due to IHC evaluation), making interpretation challenging. Moreover, hot tumors in UM might correspond to cold tumors in other cancer types owing to the inherently low mutational burden in UM. Another limitation of this study is that biopsies were more frequently performed on patients with a long treatment history and, consequently, prolonged OS. As a result, our group primarily consists of patients who have already demonstrated good OS. This potentially leads to selection bias compared with other studies, and the small number of events in our cohort restricts the feasibility of conducting a reliable multivariate Cox proportional hazards analysis. Including several covariates would have violated the recommended events-per-variable rule, resulting in potentially unstable and unreliable estimates. Another limitation of this study is that the findings are purely correlative, with no direct investigation of the underlying mechanisms involved, and it has a small cohort size, which restricts statistical power and may affect the generalizability of the findings to broader patient populations. Further, integrating NanoString and IHC data was not feasible, and the reported associations should be regarded as exploratory and hypothesis-generating rather than conclusive.

## 4. Materials and Methods

Patients were included on the basis of the availability of formalin-fixed, paraffin-embedded (FFPE) tissue samples and corresponding clinical data. Tissue samples of metastatic UM were collected from the Institute of Pathology (Uniklinikum Erlangen). Diagnoses were confirmed by rereview of the histologic slides. Clinical data and the treatment outcomes of interest were extracted from the original patient records (Uniklinikum Erlangen) and merged into a central database before analysis.

According to the RECIST 1.1 criteria (a standardized method used in clinical research to evaluate how tumors respond to treatment), all patient samples were divided into two clearly defined groups depending on how the patient responded to ICB therapy (see baseline characteristics) [44]. Group A included patients who demonstrated a clinical benefit from ICB treatment, defined as those who achieved complete response (CR), partial response (PR), stable disease (SD), or mixed response (MR). This means the disease either improved, remained stable, or showed a combination of both, but did not clearly progress. In contrast, group B comprised patients who showed no clinical benefit, characterized by progressive disease (PD) as their best overall response.

**Figure 4:**
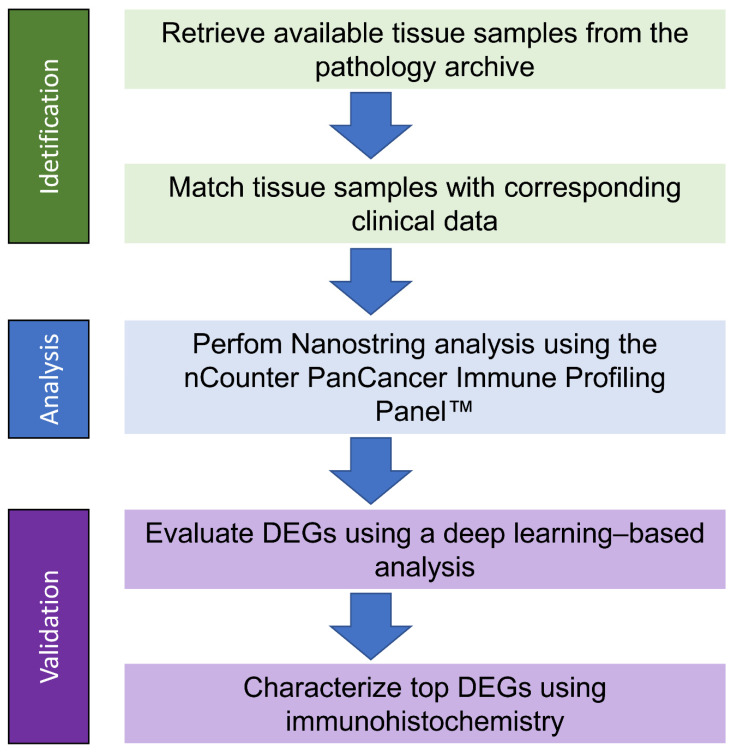
Flowchart illustrating the steps involved in the process.

For a comprehensive overview of all the work steps, see Figure 4.

### 4.1. RNA Isolation

For the isolation of total RNA, 10 µm thick sections were obtained from the FFPE blocks. The sections were microscopically examined, and only the tumor core was collected and assembled through microdissection. Afterwards, the tissue was deparaffinized, and the total RNA was isolated through the Recover All Total Nucleic Acid Isolation Kit for FFPE (Thermo Fisher Scientific, Waltham, MA, USA) according to the manufacturer’s instructions. The total RNA concentration was measured via a NanoDrop 2000c (Thermo Fisher Scientific, Waltham, MA, USA).

### 4.2. NanoString Analysis

We conducted an analysis of 770 genes, including 20 housekeeping genes for reference, associated with the immune response in cancer facilitated by the nCounter PanCancer Immune Profiling Panel™ (human) by NanoString (XT-CSO-HIP1-12). Using 200–300 ng of total RNA, we followed the manufacturer’s guidelines for sample hybridization. Detection and analysis were carried out via an nCounter^®^ Digital Analyzer. The nSolver™ 4.0 analysis software was employed for raw data processing, quality control (QC), and normalization. The QC and normalization procedures included ensuring an imaging QC of >95% field of view registration, binding density QC within the 0.1–2.25 range, positive control linearity QC with R2 above 0.95, and setting the positive control limit of detection at 0.5 fM above the mean of the negative controls plus 2× standard deviations.

### 4.3. Clinical Data

The clinical data included date of diagnosis, date of metastasis (stage IV), date of death or last contact, and demographics such as sex, age, number, and sites of metastases. Furthermore, we recorded the types of treatments and dissected ICB regarding the best radiological response evaluation on the basis of the RECIST criteria version 1.1, start date of therapy, and time to progression. The best radiologic response to ICB treatment, which was achieved during the disease course, was assessed by the site investigators and indicated as CR, PR, SD, MR, or PD. All patients were treated until disease progression or the development of unacceptable toxicity. The OS was calculated as the time from the start of ICB therapy until melanoma-specific or treatment-related death. Time-to-event analyses were performed where death or progression was considered an event. If neither occurred or if patients were lost to follow-up, the date of the last documented contact was used as a censored observation. Patients were then retrospectively assigned to groups based on the best overall clinical response observed during ICB treatment.

### 4.4. Machine Learning Pipelines

A detailed description can be found in Supplementary Materials S3. In summary, this exploratory analysis was conducted to examine gene expression patterns from a machine learning perspective as a complement to our primary statistical analysis. The data were normalized via a per-sample normalization strategy based on the Nanostring^®^ Gene Expression Data Analysis guidelines. The normalized data were divided into training (70%) and testing (30%) sets using stratified random sampling to maintain class balance. Two separate exploratory pipelines were evaluated: one using the top 5 differentially expressed genes (identified from the full dataset, introducing look-ahead bias), and one using all genes without preselection. In both pipelines, Recursive Feature Elimination (RFE) was applied exclusively to the training data to select two features, with both linear (Logistic Regression) and non-linear (Gradient Boosting Classifier) RFE methods compared to explore different feature selection patterns [10]. For each RFE approach, seven classification algorithms were compared using stratified 3-fold cross-validation on the training set, with model selection based on mean accuracy across folds. The best-performing model from each comparison was then retrained on the entire training set and evaluated on the held-out test set. Performance was assessed using accuracy, AUC-ROC, precision, recall, F1 score, and confusion matrices. No hyperparameter optimization was performed; a maximum tree depth of 3 was pre-specified for the Gradient Boosting Classifier trees to reduce overfitting risk given the small sample size. Partial dependence plots were generated for interpretability.

### 4.5. Immunohistochemistry

The tissue samples were stained via specific antibodies and protocols to assess the expression of TIGIT (T cell immunoreceptor with Ig and ITIM domains), Indolamin-2,3-Dioxygenase (IDO), and CD28. For TIGIT, the monoclonal antibody TG1 (OncoDianova, DIA-TG1, Hamburg, Germany) was used. Pretreatment was performed with TRS6 for 15 min, and the antibody was diluted 1:100. Detection was carried out via the Vectastain Elite ABC Kit, Peroxidase (PK-6100, Vector, Newark, NJ, USA), and the staining was visualized with the PermaBlue chromogen (K063, Diagnostic BioSystems, Pleasanton, CA, USA). For IDO, a polyclonal antibody (HPA 023149-100, Sigma Aldrich, Darmstadt, Germany) was applied. The pretreatment involved citrate buffer for 1 min, with an antibody dilution of 1:100. Detection was achieved via the ZytoChem Plus (AP) Polymer Kit (POLAP-006, Zytomed, Berlin, Germany), and staining was developed via the Permanent AP-Red Kit (ZUC001-125, Zytomed, Berlin, Germany). For CD28, the monoclonal antibody RM404 (NBP2-89091, NovusBio, Centennial, CO, USA) was utilized. The pretreatment consisted of citrate buffer for 1 min, and the antibody was diluted 1:1000. Staining was performed via the ZytoChem Plus (AP) Polymer Kit (POLAP-006, Zytomed, Berlin, Germany), with Permanent AP-Red (ZUC001-125, Zytomed, Berlin, Germany) used as the chromogen, resulting in red staining.

Afterward, the samples were digitized via a Pannoramic 1000 Digital Scanner (3DHISTECH Ltd., Budapest, Hungary). A Plan-Apochromat objective with 20× magnification was used to capture images with a resolution of 0.243 micrometers per pixel, resulting in an output resolution of 41.1.

The digitized slides were blindly evaluated with the help of the IRS by a board-certified pathologist [45]. The IRS is a semiquantitative evaluation method and ranges from 0 to 12. It is calculated as the product of two components: the score for the proportion of positively stained cells (0–4) and the score for staining intensity (0–3). For further details, see Figure S3.

### 4.6. Statistical Analysis

Differential gene expression analysis was performed in R (v.4.4.1) via the DESeq2 package (v.1.44.0) [46]. Positive/negative controls were excluded from the analysis, and count data were normalized using size factors estimated for housekeeping genes. The design formula adopted for the analysis was ~ICB_response, where the factor ICB_response represented the clinical outcome groups as mentioned before. Prior to selecting the final model, multiple alternative design formulas were tested to assess the influence of potential variables.

Fold change shrinkage was applied via the “ashr” method, which is integrated within the DESeq2 package, to increase the stability of the differential expression estimates [47]. Genes with a Benjamini–Hochberg adjusted *p* value less than 0.05 were considered significantly differentially expressed.

For data visualization purposes, variance-stabilizing transformed (VST) counts were generated via the vst function from DESeq2 and used to produce principal component analysis (PCA) plots, sample correlation heatmaps, and DEG heatmaps. Heatmaps illustrating either gene expression profiles or intersample correlations were generated via the pheatmap package (v.1.0.12), whereas volcano plots were produced via the EnhancedVolcano package (v.1.22.0) [48]. To investigate the biological relevance of the differentially expressed genes, functional enrichment analysis was performed via the enrichGO function from the clusterProfiler package (v.4.12.6), with a focus on the Gene Ontology (GO) biological process ontology [49]. The input for enrichment analysis consisted of gene symbols corresponding to DEGs with an adjusted *p* value < 0.05 (Benjamini–Hochberg correction). As background (universe), the full set of genes tested for differential expression (i.e., those with available adjusted *p* values in the DESeq2 results) was used. GO terms (biological process ontology) were considered significantly enriched if their adjusted *p* value was less than 0.05.

To visualize functional relationships among enriched GO terms, similarity between terms was calculated via the pairwise_termsim() function of the clusterProfiler package. The resulting similarity matrix was converted into a distance matrix, which was then used as input for dimensionality reduction via the umap() function from the umap package (v.0.2.10.0). The resulting embeddings were clustered via the Ward method. D Hierarchical clustering into five functional clusters (k = 5), enabling the identification of major biological themes across enriched pathways.

The survival and progression probabilities were estimated via the Kaplan–Meier method. To test for a significant moderating factor, the survival curves were compared with the log-rank test. Levene’s test was used to assess the equality of continuous variables across the different groups. If the *p* value was < 0.05, indicating unequal variances, a Welch’s t-test was performed; otherwise, Student’s t-test was performed. Pearson’s Chi-square tests were conducted to assess group differences in categorical variables. If the assumptions of the Chi-square test were violated (e.g., low expected cell frequencies), Fisher’s exact test was applied instead. A two-sided *p* value of < 0.05 was used to define statistical significance. Statistical analyses were performed via IBM^®^ SPSS Statistics (version 28.0.0.0; Armonk, NY, USA).

## 5. Conclusions

Our analyses revealed that patients who respond well to ICB have tumors with upregulated genes associated with an active immune response, as seen in the NanoString^®^ data. These findings suggest that ICB is most effective in tumors that already exhibit an immune response rather than in immune-cold tumors. IHC analysis further identified CD28 expression on immune cells and the machine learning pipeline CCL8 as potential candidates for response prediction. Given the exploratory nature of our findings, CD28 and CCL8 should currently be regarded as investigational markers that require analytical and clinical validation before they can be considered as biomarkers.

## Figures and Tables

**Table 1:** Characteristics of the study samples.

	Categories	Group A (*n* = 16)	Group B (*n* = 14)	*p* Value
Therapy response to ICB	CR	0 (0%)	N/A	N/A
	PR	5 (31.25%)	N/A	
	SD	10 (62.5%)	N/A	
	MR	1 (6.25%)	N/A	
	PD	N/A	14 (100%)	
Age (date of diagnosis of Stage IV)	Mean in years	62.8	60.5 years	0.64
		Std. dev. = 14.1	Std. dev. = 12	
Sex	Male	7 (43.75%)	11 (78.6%)	0.052
	Female	9 (56.25%)	3 (21.4%)	
LDH (date of diagnosis of Stage IV)	Mean in U/l	239.4	271.5	0.37
		Std. dev. = 68.32	Std. dev. = 116.24	
	N/A	1 (6.25%)	0 (0%)	
ECOG status (date of diagnosis of Stage IV)	0	13 (81.25%)	13 (92.9%)	0.53
	1	2 (12.5%)	1 (7.1%)	
	N/A	1 (6.25%)	0 (0%)	
Sites of metastases (*n*)	Liver only	2 (12.5%)	2 (14.3%)	0.33
	Liver and Extrahepatic	10 (62.5%)	11 (78.6%)	
	Extrahepatic only	4 (25%)	1 (7.1%)	
Biopsy timing (*n*)	Before systemic therapy	10 (62.5%)	9 (64.3%)	0.91
	After systemic therapy	6 (37.5%)	5 (35.7%)	
NanoString cartridges (*n*)	Batch 1	4 (25%)	3 (21.4%)	0.33
	Batch 2	7 (43.75%)	3 (21.4%)	
	Batch 3	1 (6.25%)	4 (28.6%)	
	Batch 4	4 (25%)	4 (28.6%)	
Biopsy location	Liver	10 (62.5%)	10 (71.4%)	0.71
	Extrahepatic	6 (37.5%)	4 (28.6%)	
Overall survival	Median in month	48.9	15.8	<0.001 Log Rank (Mantel–Cox)
	95% Confidence Interval	27.3–70.4	0–35.3	

**Table 2:** Immunoreactivity scores.

	Group	N	Mean	Std. Deviation	*t* Test (One-Sided)
TIGIT	A	15	1.6	0.95	*p* = 0.4
	B	13	1.5	1.39	
CD28	A	14	2.6	2.09	*p* = 0.039
	B	14	1.1	1.81	
IDO-1	A	16	0.75	1	*p* = 0.175
	B	12	0.42	0.78	

## Data Availability

The original contributions presented in this study are included in the article/Supplementary Material. Further inquiries can be directed to the corresponding author.

## Supplementary Materials

The following supporting information can be downloaded at: https://www.mdpi.com/article/10.3390/ijms26209964/s1.

## Abbreviations

The following abbreviations are used in this manuscript:

CR	complete response
DCB	double checkpoint blockade
EMA	European Medicines Agency
FDA	Food and Drug Administration
FFPE	formalin-fixed, paraffin-embedded
GO	Gene Ontology
ICB	immune checkpoint blockade
IDO	Indolamin-2,3-Dioxygenase
IRS	immunoreactive score
LBCL	large B cell lymphoma
MR	mixed response
ORA	overrepresentation analysis
OS	overall survival
PCA	principal component analysis
PD	progressive disease
PR	partial response
QC	quality control
RFE	recursive feature elimination
SD	stable disease
TAM	tumor-associated macrophages
tebe	tebentafusp
TIGIT	T cell immunoreceptor with Ig and ITIM domains
TILs	tumor-infiltrating lymphocytes
UM	Uveal melanoma
VST	variance-stabilizing transformed
