# Supplementary material for: Checkpoint Blockade Efficacy in Uveal Melanoma Is Linked to Tumor Immunity, CD28, and CCL8
Source: Int J Mol Sci. 2025 Oct 13;26(20):9964. doi: 10.3390/ijms26209964 (PMC12564235; doi:10.3390/ijms26209964)
Supplement: Supplementary file 1 [file ijms-26-09964-s001.zip › Supplementary Figures S1-S3.pdf]

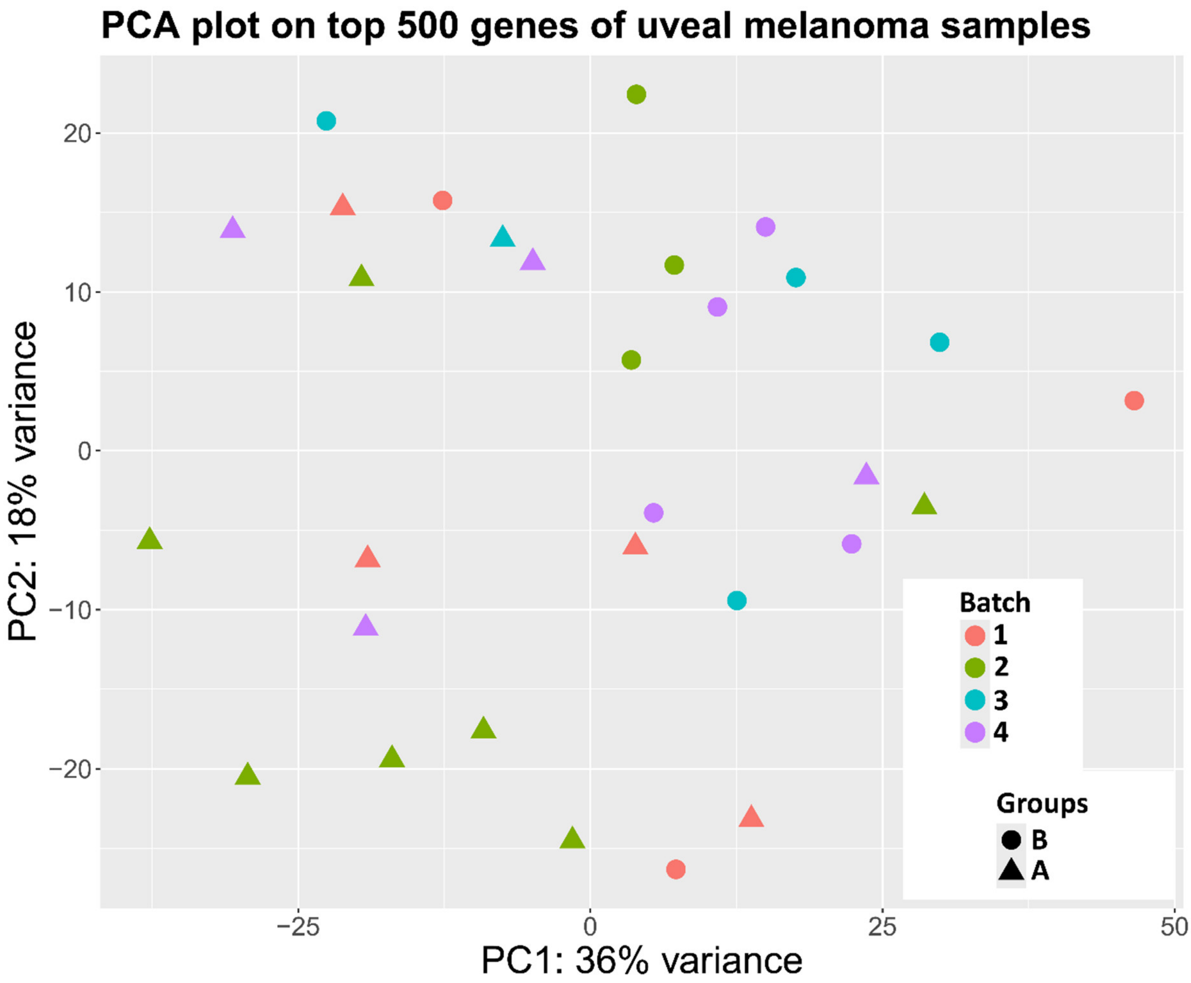


**Figure S1:** Principal component analysis (PCA) plot of uveal melanoma samples based on the top 500 most variable genes. The plot shows the first two principal components and the associated explained variance. The color corresponds to the NanoString® cartridges, and the shape corresponds to the different groups.

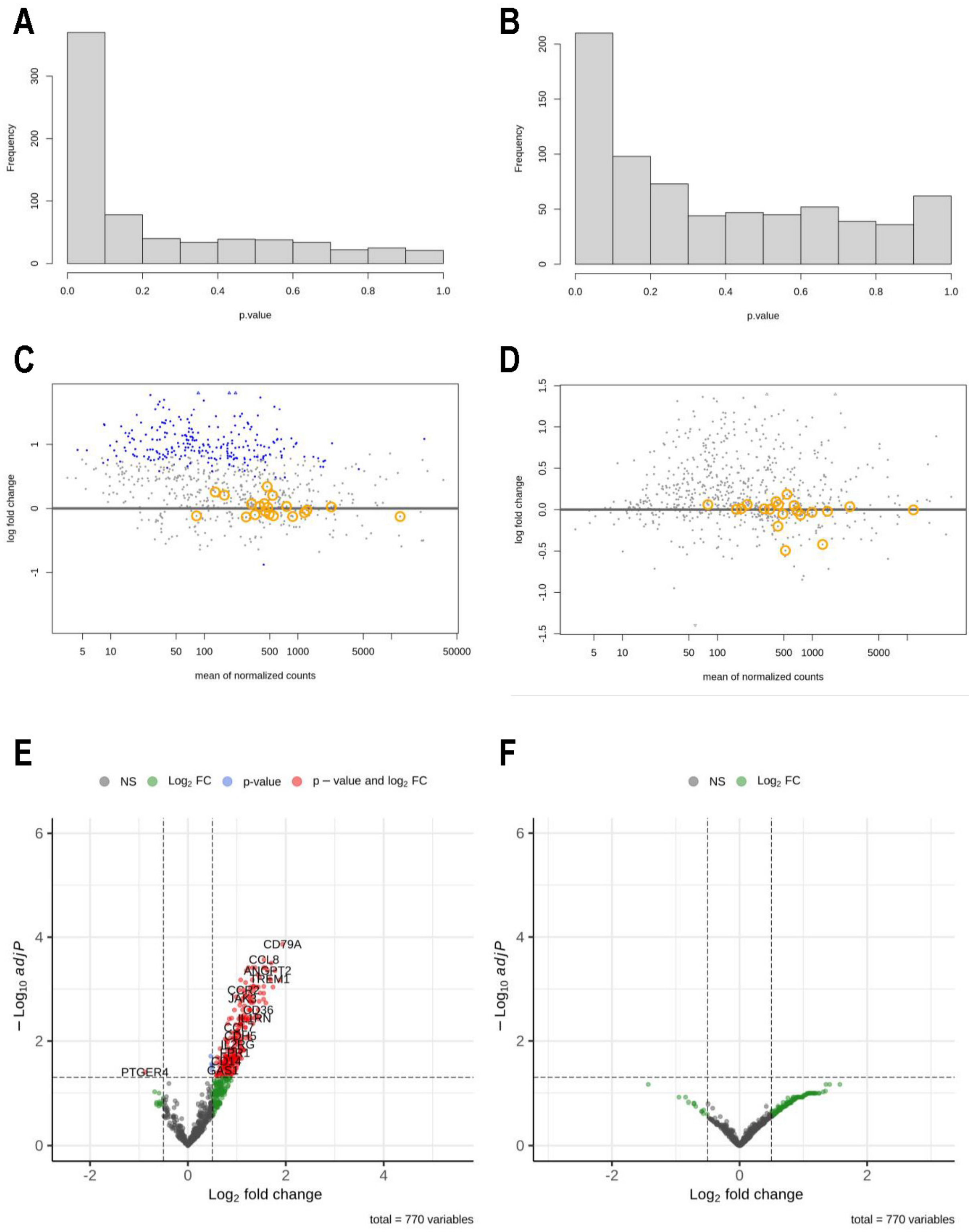


**Figure S2:** The figure separately shows comparisons of responders (group A) vs. non-responders (group B) using only baseline samples (A, C, E; n=19) and only longitudinal samples (B, C, F; n=11). A and B: Histograms showing the distribution of p-values across analysed. The x-axis represents p-value ranges from 0 to 1, while the y-axis indicates the count of features within each bin. C and D: MA plots illustrating differential gene expression with highlighted housekeeping genes. Differentially expressed

genes (p.adjust < 0.05) are highlighted in blue, indicating statistically significant changes in expression levels between conditions. Housekeeping genes are encircled in yellow, serving as internal controls. Non-highlighted points represent genes not meeting criteria for differential expression. E and F: Volcano plots of the differential gene expression analysis comparing Group A (PR, SD, MR) to Group B (PD). Genes are colored based on the following cutoffs: log2 fold change (Log2FC) > 0.5 and adjusted p-value (P.adjust) < 0.05. Gray: non-significant genes (NS), green: genes with Log2FC > 0.5 only, blue points: genes with adjusted p-value < 0.05 only, and red: genes meeting both criteria (Log2FC > 0.5 and adjusted p-value < 0.05). Interestingly, the baseline samples showed results that were largely consistent with the main analysis (see list of DEGs provided in the supplementary material 3), whereas no DEGs were identified in the longitudinal samples, which further supports the validity and specificity of the main results.

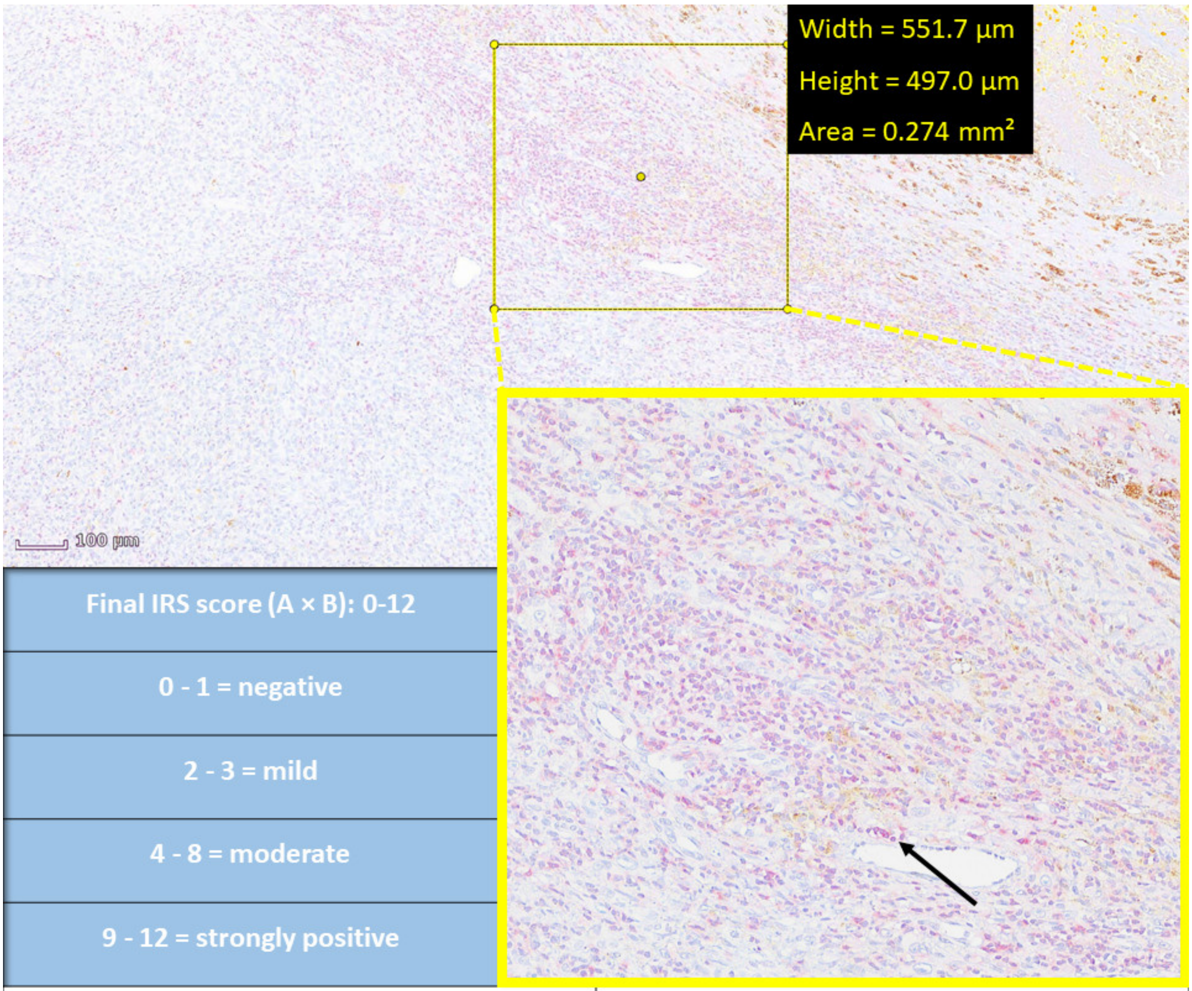


| **A) Percentage of positive cells** | **B) intensity of staining** |
|---|---|
| 0 = no positive cells | 0 = no reaction |
| 1 = <10% positive cells | 1 = mild reaction |
| 2 = 10-50% positive cells | 2 = moderate reaction |
| 3 = 51-80% positive cells | 3 = intense reaction |
| 4 = >80% positive cells | **Final IRS score (A × B): 0-12** |

**Figure S3:** This image shows CD28 staining of a liver metastasis from uveal melanoma. The black arrow highlights an area of CD28 immunoreactivity within the perivascular lymphocytic infiltrate. To assess the expression levels of potential biomarkers (CD28 and IDO1) in the tumor tissue, the immunoreactive score (IRS) was applied as a semiquantitative evaluation method, allowing for a systematic and comparative analysis of the staining intensity and distribution.
