# Supplementary material for: Checkpoint Blockade Efficacy in Uveal Melanoma Is Linked to Tumor Immunity, CD28, and CCL8
Source: Int J Mol Sci. 2025 Oct 13;26(20):9964. doi: 10.3390/ijms26209964 (PMC12564235; doi:10.3390/ijms26209964)
Supplement: Supplementary file 1 [file ijms-26-09964-s001.zip › Supplementary Materials S3.pdf]

Supplementary Material 1: Machine Learning Analysis

Methods and Results

Preamble: purpose and scope of this analysis

This supplementary material provides comprehensive methodological details and complete results for the exploratory machine learning analysis described in sections 2.4 and 4.4 of the manuscript.

Given the small sample size (N=30), this analysis was designed as an exploratory and descriptive complement to our primary statistical analysis, rather than as a definitive predictive modeling effort. Machine learning provides a complementary lens to differential expression analysis by examining multivariate patterns and gene combinations that may jointly contribute to treatment response prediction.

1. Overview of Analysis Approaches

We conducted four separate machine learning pipelines that differ in two dimensions: (1) the initial gene set used as input, and (2) the algorithm used for recursive feature elimination (RFE). Table S1 summarizes these four approaches.

Table S1. Summary of Machine Learning Pipelines

Pipeline	Initial Gene Set	RFE Method	Purpose
**1**	Top 5 DEGs from full dataset (IDO1, CD28, CCL8, CXCR3, TIGIT)	Logistic Regression	Explore genes of primary interest using linear feature selection
**2**	Top 5 DEGs from full dataset (IDO1, CD28, CCL8, CXCR3, TIGIT)	Gradient Boosting Classifier	Explore genes of primary interest using non-linear feature selection
**3**	All genes (no preselection)	Logistic Regression	Unbiased exploration with linear feature selection
**4**	All genes (no preselection)	Gradient Boosting Classifier	Unbiased exploration with non-linear feature selection

Pipelines 1 & 2 (Preselected genes) were designed to focus on genes already identified as differentially expressed in our primary statistical analysis. By constraining the analysis to these biologically relevant candidates, we sought to understand how machine learning algorithms would prioritize and combine these known biomarkers. However, because the differential expression analysis that generated this gene list used the full dataset (including samples that later became the test set), these pipelines involve look-ahead bias and cannot be used for reliable predictive performance estimation. Results from these pipelines are purely descriptive.

Pipelines 3 & 4 (All genes) follow standard machine learning best practices by performing feature selection exclusively on the training data, without any prior knowledge of differential expression results. This approach avoids look-ahead bias in feature selection and provides a more rigorous assessment of potential predictive performance, though still limited by the small sample size.

Common methodology across all pipelines:

Despite differences in initial gene sets and RFE algorithms, all four pipelines followed an identical workflow:

1. Normalization of gene expression data (sample-wise, to prevent data leakage)

2. Stratified train-test split (70/30)
3. RFE on training data only to select 2 genes
4. Comparison of 7 classification models via 3-fold cross-validation on training data
5. Selection of best-performing model based on mean cross-validation accuracy
6. Final model training on entire training set
7. Evaluation on held-out test set

The manuscript (Section 2.4) reports results from two pipelines:

- **Pipeline 2:** Selected CCL8 and TIGIT; achieved ~78% test accuracy with a Logistic Classifier
- **Pipeline 3:** Selected CASP9 and HLA-DRB1; achieved ~89% test accuracy with a Logistic Classifier

Complete results for all four pipelines are provided in Section 8 of this supplementary material.

2. Normalization

The raw Nanostring counts were normalized sample-wise following the Nanostring Gene Expression Data Analysis guidelines. The raw counts were transformed into normalized counts in the following steps for each sample:

1. The mean of the negative controls was subtracted from the raw counts. If the counts became negative, they were set to zero.

$$x^1_{ig} = max\left(0,\ x_{ig} - \frac{1}{|NEG|}\sum_{g\in NEG} x_{ig}\right)$$

2. The background-corrected gene expression was divided by the geometric mean of the positive controls. A +1 was added to prevent division by zero, or by a very small number. The formula is expressed in terms of the exponential function and the arithmetic mean here

$$x_{ig}^{2} = x_{ig}^{1} / exp\left(\frac{1}{|POS|} \sum_{g \in POS} log\left(x_{ig}^{1} + 1\right)\right)$$

3. The background-corrected gene expression was divided by the geometric mean of the housekeeping genes. A +1 was added to prevent division by zero, or by a very small number. The formula is expressed in terms of the exponential function and the arithmetic mean here

$$x_{ig}^{3} = x_{ig}^{2} / exp\left(\frac{1}{|HK|} \sum_{g \in HK} log\left(x_{ig}^{2} + 1\right)\right)$$

4. The results were finally log-transformed.

$$\overline{x_{ig}} = log\left(x_{ig}^{3} + 1\right)$$

The normalization procedure corresponds to the standard one where all the cross-sample terms have been removed. This ensures there is no data-leakage in the normalization process, which is a prophylaxis needed to ensure meaningful results in Machine Learning. Another possibility could have been to estimate the cross-sample terms for the training dataset, and impute this transformation to the test set, however, we went for this simple approach due to its simplicity.

In the formulae, x_{ig}^{k} refers to the level of expression of sample *i* and gene *g* on normalization step k, with an overline indicating the final normalized gene expression. *POS*, *NEG* and *HK* refer to the Nanostring-given sets of positive controls, negative controls and housekeeping genes respectively. | · | refers to the number of genes in a given set, and *log* refers to the natural logarithm.

3. Seed setting and computational setup

To ensure the reproducibility of our results and minimize variability due to non-deterministic operations, we set a fixed random seed and we control the number of threads used by various libraries that may use parallelism. The environment variables `OMP_NUM_THREADS`, `OPENBLAS_NUM_THREADS`, `MKL_NUM_THREADS`, `VECLIB_MAXIMUM_THREADS`, and `NUMEXPR_NUM_THREADS` are all set to "1" to limit these libraries to a single thread, which helps avoid performance discrepancies or variations due to multithreaded computations. This

configuration ensures that the computation is deterministic and consistent across different runs, facilitating reproducibility and fair comparisons of results.

```
seed = 440636
random.seed(seed)
np.random.seed(seed)

os.environ['PYTHONHASHSEED'] = str(seed)
os.environ["OMP_NUM_THREADS"] = "1"
os.environ["OPENBLAS_NUM_THREADS"] = "1"
os.environ["MKL_NUM_THREADS"] = "1"
os.environ["VECLIB_MAXIMUM_THREADS"] = "1"

os.environ["NUMEXPR_NUM_THREADS"] = "1"
```

4. Train-test split

In accordance with Machine Learning methodologies, we divided the dataset into two subsets: training and testing. The size of the training set was set to 70% of the original size, with the remaining 30% corresponding to the testing set. In order to ensure–as much as possible–a similar distribution of the covariates among the samples belonging to the two groups, a *"combined category"* was created by concatenating the following covariates: *Sex, Cohort, Batch, Biopsy Location* and our clinical outcome of interest, namely, whether the patients showed progressive disease or whether their illness was stable or responded to therapy in some way; this target variable is known internally as *grouping_2* in our code. The samples whose combination of clinical covariates was rarer than 5 were considered "rare", and therefore set to "Other". The splitting was then performed stratifying by this category, this is, by trying to keep the same proportion of each subgroup of the *combined category* in both training and testing.

5. Feature selection

Given the high number of genes with respect to the number of samples, we needed to constrain the number of genes to obtain a sensible model [1], [2]. We selected the features for the models by *Recursive Feature Elimination (RFE).* Recursive Feature Elimination relies on training the model and removing the least important feature iteratively until one obtains a subset of a desired size; it has already been used for gene subselection [1]. We used two different models as the base model for this process: a Logistic Regression and a Gradient

Boosting Classifier [3], to capture linear and non-linear patterns. We performed this RFE either on all genes, or on a selected subset of them. We chose to keep 2 genes after the RFE, to follow a usual rule-of-thumb in machine learning of roughly 10 samples per feature.

6. Model comparison and cross-validation

Seven models were compared on the training dataset by performing 3-fold stratified cross-validation, with *grouping_2* being the stratifying factor. The dataset was therefore divided into 3 splits, and the models were repeatedly trained on 2 of those splits, and tested on the remaining split 3 times. For each test of the model, the accuracy was calculated. In the end, the model with the highest average *accuracy* was selected as the best performing model.

The models chosen for the comparison were: Logistic Regression, Lasso Classifier (Logistic Regression with *l1* regularization); Support Vector Classifiers with three different kernels: linear, radial basis function (RBF), polynomial, and sigmoid; and Category Boosting Classifier. The choice of these models follows the desire to explore both linear and non-linear patterns in the data, our capacity to ensure replicability with each run and availability and ease of use in *Python.* Each model included standardization as a prior step to the training and predicting.

Logistic Regression:

- `solver='liblinear'`: This solver is used because it supports L1 regularization.
- `random_state=seed`: A fixed random seed ensures reproducibility of results, eliminating randomness in training.
- `n_jobs=1`: Limits the computation to a single core.

Lasso Classifier (Logistic Regression with L1 penalty):

- `penalty='l1'`: This specifies L1 regularization, which performs feature selection by shrinking less important coefficients to zero. It is particularly useful for handling multicollinearity by reducing the effect of correlated features.
- `solver='liblinear'`: This solver is used because it supports L1 regularization.
- `random_state=seed`: A fixed random seed ensures reproducibility of results, eliminating randomness in training.
- `n_jobs=1`: Limits the computation to a single core.
-

Support Vector Classifier with RBF Kernel (SVCrbf):

- `kernel='rbf'`: The radial basis function (RBF) kernel is used to allow the model to capture non-linear relationships between features.

- `probability=True`: This enables probability estimates for each class, which are useful for model metrics.
- `random_state=seed`: Ensures replicability.

Support Vector Classifier with Polynomial Kernel (SVCpoly):

- `kernel='poly'`: The polynomial kernel is used to find decision boundaries that are polynomial functions of the input features, capturing more complex non-linear relationships.
- `probability=True`: Like the RBF SVC, this allows the model to output class probabilities, used for model metrics.
- `random_state=seed`: Ensures replicability.

Support Vector Classifier with Sigmoid Kernel (SVCsig):

- `kernel='sigmoid'`: The sigmoid kernel is based on the hyperbolic tangent function and is another way to map data to higher-dimensional space, capturing non-linear relationships.
- `probability=True`: Like to the previous SVCs, this allows the model to output class probabilities, used for model metrics.
- `random_state=seed`: Ensures replicability.

Support Vector Classifier with Linear Kernel (SVM Linear):

- `kernel='linear'`: Uses a linear kernel to model a hyperplane that separates the classes.
- `probability=True`: Like the other SVM variants, this allows the model to output class probabilities, used for model metrics.
- `random_state=seed`: Guarantees that the results are reproducible by fixing the random initialization.

CatBoost Classifier:

- `max_depth=3`: Limits the depth of individual trees in the gradient boosting model, which helps prevent overfitting and ensures a more generalizable model. 3 is usually the default max_depth set for other similar tree-based algorithms.
- `verbose=0`: Disables output during training.
- `random_seed=seed`: Ensures reproducibility.
- `thread_count=1`: Limits the computation to a single thread, ensuring reproducibility.

- `random_strength=0`: Controls the amount of randomness in the model. Setting it to 0 means no additional randomness is introduced during the training process, ensuring a deterministic result.

7. Performance Evaluation Metrics

To comprehensively assess model performance, we calculated multiple classification metrics using scikit-learn implementations.

7.1. Standard Classification Metrics

Accuracy: Proportion of correct predictions among all predictions: (TP + TN) / (TP + TN + FP + FN)

AUC-ROC: Area under the Receiver Operating Characteristic curve. Measures discriminative ability independent of classification threshold. Values range from 0.5 (chance) to 1.0 (perfect discrimination).

Precision: Proportion of true positives among positive predictions: TP / (TP + FP).

Recall (Sensitivity): Proportion of actual positives correctly identified: TP / (TP + FN).

F1 Score: Harmonic mean of precision and recall: 2 × (Precision × Recall) / (Precision + Recall).

Confusion Matrix: 2×2 table showing counts of true positives (TP), true negatives (TN), false positives (FP), and false negatives (FN), from which all other metrics are derived.

7.2. Bootstrap Confidence Intervals

Given the small test set size (n=9), we estimated uncertainty in performance metrics using stratified bootstrap resampling with 1,000 iterations. In each iteration:

1. Samples were drawn with replacement from each class separately to maintain class balance
2. All metrics were calculated on the resampled data
3. The 95% confidence interval was estimated as the 2.5th and 97.5th percentiles of the 1,000 bootstrap values

This stratified approach ensures both classes are represented in each resample, preventing undefined metrics (particularly AUC-ROC, which requires both classes present). Bootstrap methods are well-suited for small samples as they make minimal distributional assumptions, though the wide confidence intervals reflect the fundamental uncertainty inherent in estimating performance from limited data. Implementation used NumPy and our fixed-seed setup ensured replicability.

7.3. Cross-Validation Metrics

During model selection, we used 3-fold stratified cross-validation on the training set, reporting for each model:

- **Mean accuracy:** Average across the 3 folds
- **Standard deviation:** Variability across folds, indicating model stability
- **Min/Max accuracy:** Range of performance across folds

High variance across folds may indicate overfitting, while consistent performance suggests stability.

8. Results

This section presents the complete performance metrics for all four machine learning pipelines. For each pipeline, we report: (1) the genes selected by RFE, (2) cross-validation performance of all seven candidate models on the training set, (3) final test set performance of the best model, and (4) classification metrics with bootstrap confidence intervals.

8.1. Pipeline 1: Preselected Genes (Top 5 DEGs) + Logistic Regression RFE

Initial genes: IDO1, CD28, CCL8, CXCR3, TIGIT

Genes selected by RFE: CD28, CCL8

8.1.1. Cross-Validation Performance (Training Set)

Table S2 shows the mean accuracy ± standard deviation across 3 stratified cross-validation folds for each of the seven candidate models.

Table S2. Cross-validation performance for Pipeline 1

Model	Mean Accuracy	SD	Min Accuracy	Max Accuracy
Logistic Regression	0.762	0.067	0.714	0.857
Lasso Classifier	0.667	0.067	0.571	0.714
SVC (Linear)	0.714	0.000	0.714	0.714
SVC (RBF)	0.762	0.067	0.714	0.857
SVC (Polynomial)	0.762	0.067	0.714	0.857
SVC (Sigmoid)	0.714	0.000	0.714	0.714
CatBoost	0.524	0.067	0.429	0.571

Best model by CV performance: Three models achieved equivalent mean CV accuracy (0.762 ± 0.067): Logistic Regression, SVC (RBF), and SVC (Polynomial). Logistic Regression was selected since it was the simplest and most interpretable model.

8.1.2. Test Set Performance

The Logistic Regression model was retrained on the entire training set and evaluated on the held-out test set (n = 9 samples).

Table S3. Test set performance metrics for Pipeline 1

Metric	Value	95% Confidence Interval
Accuracy	0.556	[0.333, 0.889]
AUC-ROC	0.450	[0.050, 0.850]
Precision	0.667	[0.000, 1.000]
Recall (Sensitivity)	0.400	[0.000, 0.800]

Metric	Value	95% Confidence Interval
F1 Score	0.500	[0.000, 0.889]

Confusion Matrix:

	Predicted: Progressive Disease	Predicted: Stable/Response
**Actual: Progressive Disease**	3	1
**Actual: Stable/Response**	3	2

Figure S1. ROC curve for Pipeline 1 test set predictions.

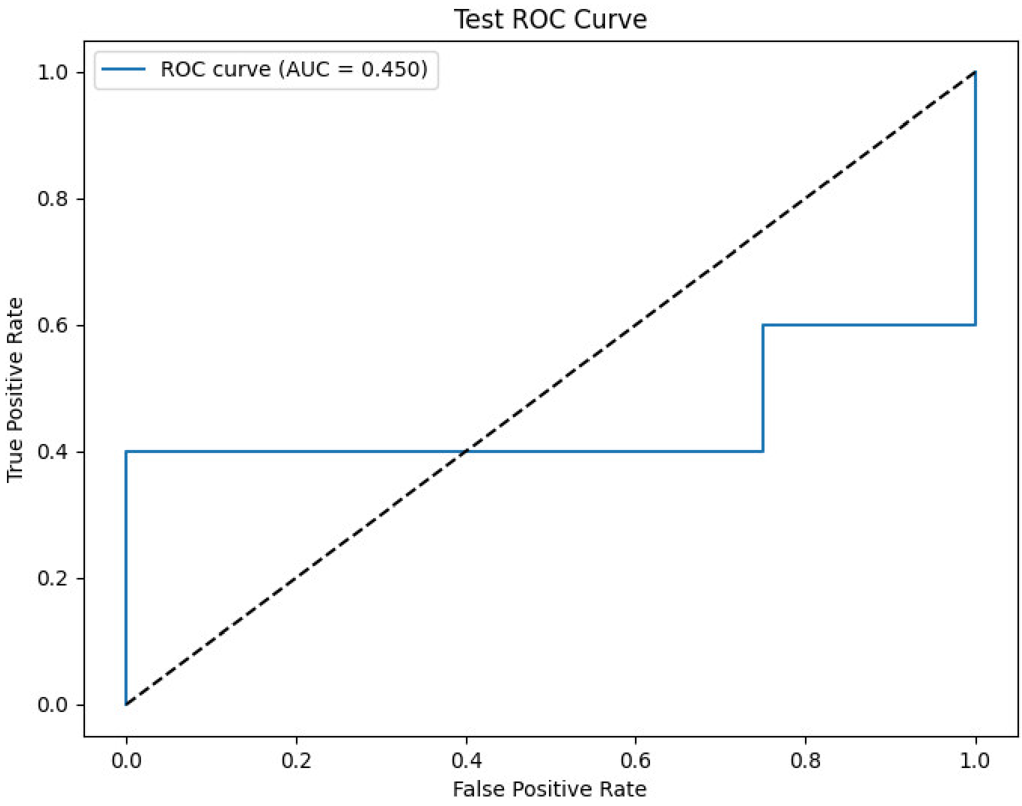

8.2. Pipeline 2: Preselected Genes (Top 5 DEGs) + Gradient Boosting Classifier RFE

Initial genes: IDO1, CD28, CCL8, CXCR3, TIGIT

Genes selected by RFE: CCL8, TIGIT *(as reported in manuscript)*

8.2.1. Cross-Validation Performance (Training Set)

Table S4. Cross-validation performance for Pipeline 2

Model	Mean Accuracy	SD	Min Accuracy	Max Accuracy
Logistic Regression	0.667	0.067	0.571	0.714
Lasso Classifier	0.571	0.117	0.429	0.714
SVC (Linear)	0.619	0.067	0.571	0.714
SVC (RBF)	0.619	0.135	0.429	0.714
SVC (Polynomial)	0.524	0.135	0.429	0.714
SVC (Sigmoid)	0.667	0.067	0.571	0.714
CatBoost	0.476	0.067	0.429	0.571

Best model by CV performance: Two models achieved equivalent mean CV accuracy (0.667 ± 0.067): Logistic Regression and SVC (Sigmoid). Logistic Regression was selected as the simplest and most interpretable model.

8.2.2. Test Set Performance

The Logistic Regression model was retrained on the entire training set and evaluated on the held-out test set (n = 9 samples).

Table S5. Test set performance metrics for Pipeline 2

Metric	Value	95% Confidence Interval
Accuracy	0.778	[0.556, 1.000]
AUC-ROC	0.700	[0.300, 1.000]
Precision	1.000	[1.000, 1.000]
Recall (Sensitivity)	0.600	[0.200, 1.000]
F1 Score	0.750	[0.333, 1.000]

Confusion Matrix:

	Predicted: Progressive Disease	Predicted: Stable/Response
**Actual: Progressive Disease**	4	0
**Actual: Stable/Response**	2	3

Figure S2. ROC curve for Pipeline 2 test set predictions.

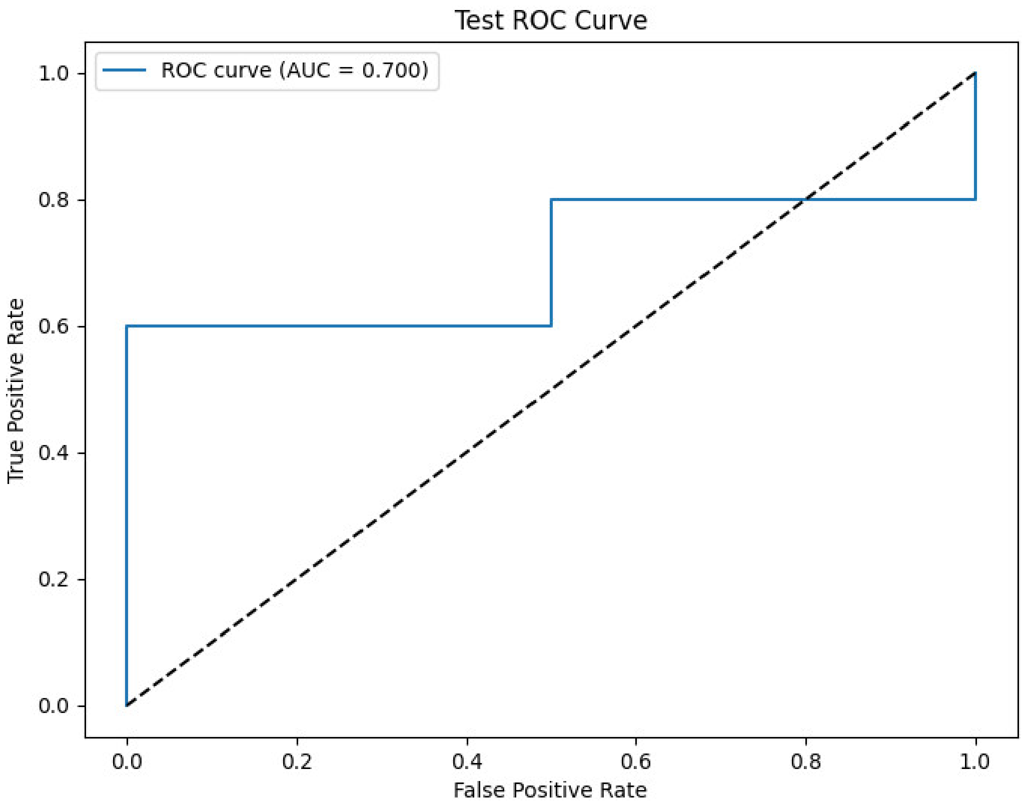

8.3. Pipeline 3: All Genes + Logistic Regression RFE

Initial genes: All genes in dataset (n = 784)

Genes selected by RFE: CASP9, HLA-DRB1 *(as reported in manuscript)*

8.3.1. Cross-Validation Performance (Training Set)

Table S6. Cross-validation performance for Pipeline 3

Model	Mean Accuracy	SD	Min Accuracy	Max Accuracy
Logistic Regression	0.810	0.135	0.714	1.000
Lasso Classifier	0.810	0.135	0.714	1.000
SVC (Linear)	0.810	0.135	0.714	1.000
SVC (RBF)	0.762	0.067	0.714	0.857
SVC (Polynomial)	0.762	0.067	0.714	0.857
SVC (Sigmoid)	0.762	0.178	0.571	1.000
CatBoost	0.762	0.067	0.714	0.857

Best model by CV performance: Three models achieved equivalent mean CV accuracy (0.810 ± 0.135): Logistic Regression, Lasso Classifier, and SVC (Linear). Logistic Regression was selected as the simplest model.

8.3.2. Test Set Performance

The Logistic Regression model was retrained on the entire training set and evaluated on the held-out test set (n = 9 samples).

Table S7. Test set performance metrics for Pipeline 3

Metric	Value	95% Confidence Interval
Accuracy	0.889	[0.667, 1.000]
AUC-ROC	0.800	[0.400, 1.000]
Precision	0.833	[0.625, 1.000]
Recall (Sensitivity)	1.000	[1.000, 1.000]
F1 Score	0.909	[0.769, 1.000]

Confusion Matrix:

	Predicted: Progressive Disease	Predicted: Stable/Response
**Actual: Progressive Disease**	3	1
**Actual: Stable/Response**	0	5

Figure S3. ROC curve for Pipeline 3 test set predictions.

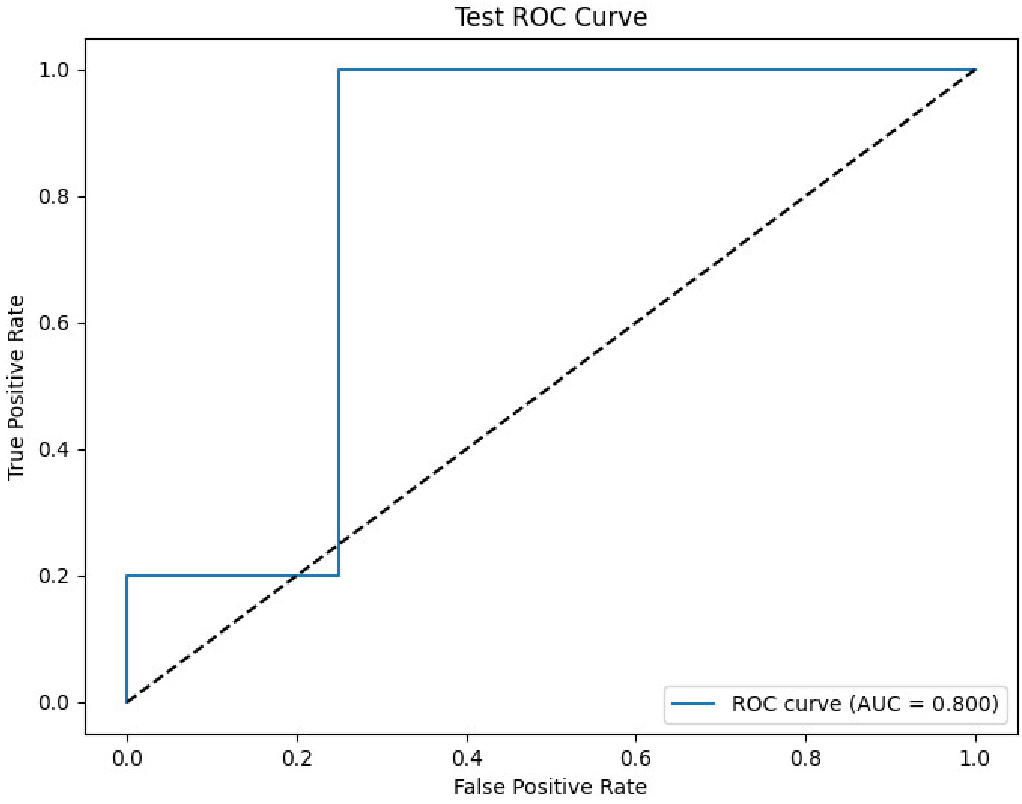

8.4. Pipeline 4: All Genes + Gradient Boosting Classifier RFE

Initial genes: All genes in dataset (n = 784)

Genes selected by RFE: DKK1, SHC2

8.4.1. Cross-Validation Performance (Training Set)

Table S8. Cross-validation performance for Pipeline 4

Model	Mean Accuracy	SD	Min Accuracy	Max Accuracy
Logistic Regression	0.333	0.178	0.143	0.571
Lasso Classifier	0.333	0.135	0.143	0.429
SVC (Linear)	0.286	0.117	0.143	0.429
SVC (RBF)	0.429	0.233	0.143	0.714
SVC (Polynomial)	0.476	0.067	0.429	0.571
SVC (Sigmoid)	0.476	0.135	0.286	0.571
CatBoost	0.429	0.117	0.286	0.571

Best model by CV performance: SVC (Polynomial) (Mean accuracy = 0.476 ± 0.067)

8.4.2. Test Set Performance

The SVC (Polynomial) model was retrained on the entire training set and evaluated on the held-out test set (n = 9 samples).

Table S9. Test set performance metrics for Pipeline 4

Metric	Value	95% Confidence Interval
Accuracy	0.556	[0.222, 0.889]
AUC-ROC	0.400	[0.000, 0.850]
Precision	0.667	[0.000, 1.000]
Recall (Sensitivity)	0.400	[0.000, 0.800]
Specificity	0.750	[0.500, 1.000]
F1 Score	0.500	[0.000, 0.889]

Confusion Matrix:

	Predicted: Progressive Disease	Predicted: Stable/Response
**Actual: Progressive Disease**	3	1
**Actual: Stable/Response**	3	2

Figure S4. ROC curve for Pipeline 4 test set predictions.

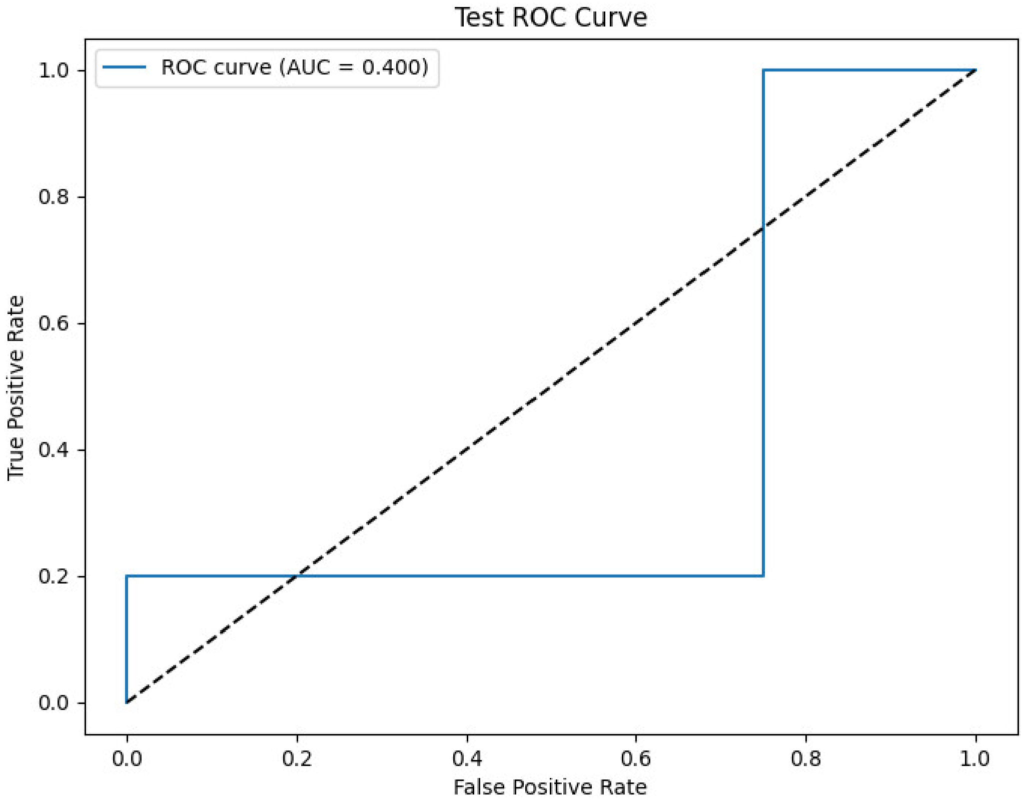
Test ROC Curve
ROC curve (AUC = 0.400)
True Positive Rate
False Positive Rate
0.0
0.2
0.4
0.6
0.8
1.0

8.5. Summary Comparison Across All Pipelines

Table S10. Test set performance comparison across all four pipelines

Pipeline	RFE Method	Gene 1	Gene 2	Best Model	Test Accuracy	Test AUC-ROC	Test F1
1	Logistic Reg	CD28	CCL8	Logistic Reg	0.556	0.450	0.500
2	Gradient Boost	CCL8	TIGIT	Logistic Reg	0.778	0.700	0.750
3	Logistic Reg	CASP9	HLA-DRB1	Logistic Reg	0.889	0.800	0.909
4	Gradient Boost	DKK1	SHC2	SVC (Poly)	0.556	0.400	0.500

9. Feature Importance and Interpretation

To understand how the selected genes influence model predictions, we generated partial dependence plots (PDPs) for the two pipelines reported in the manuscript. PDPs visualize the marginal effect of a feature on the predicted outcome, revealing whether the relationship between gene expression and treatment response is linear or non-linear, and whether higher expression is associated with increased probability of stable/response versus progressive disease.

Pipeline 2: CCL8 and TIGIT *(Preselected genes approach)*

Figure S5. Partial dependence plots for CCL8 (left) and TIGIT (right) from Pipeline 2.

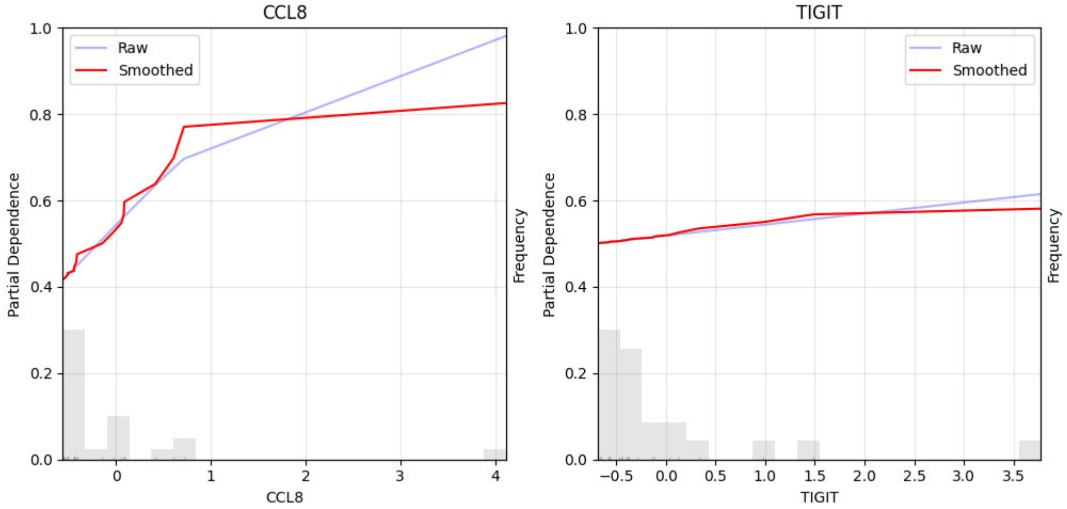

Pipeline 3: CASP9 and HLA-DRB1 *(All-genes approach)*

Figure S6. Partial dependence plots for CASP9 (left) and HLA-DRB1 (right) from Pipeline 3.

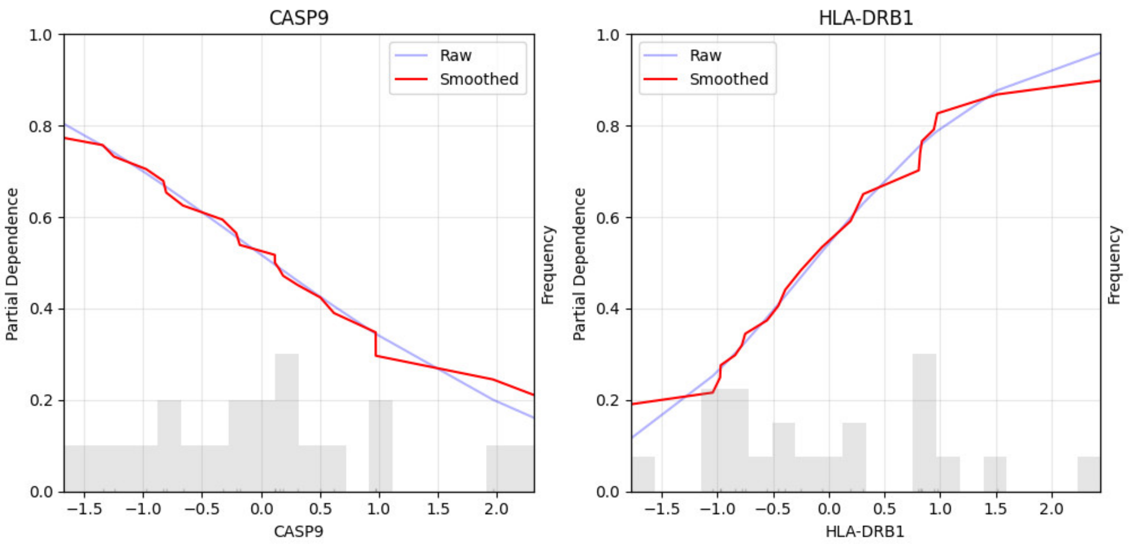

References:

[1] Guyon, Isabelle & Weston, Jason & Barnhill, Stephen & Vapnik, Vladimir. (2002). Gene Selection for Cancer Classification Using Support Vector Machines. Machine Learning. 46. 389-422. 10.1023/A:1012487302797.

[2] Berisha V, Krantsevich C, Hahn PR, Hahn S, Dasarathy G, Turaga P, Liss J. Digital medicine and the curse of dimensionality. NPJ Digit Med. 2021 Oct 28;4(1):153. doi: 10.1038/s41746-021-00521-5. PMID: 34711924; PMCID: PMC8553745.

[3] Friedman, Jerome. (2000). Greedy Function Approximation: A Gradient Boosting Machine. The Annals of Statistics. 29. 10.1214/aos/1013203451.
